# Radiomorphological signs and clinical severity of SARS-CoV-2 lineage B.1.1.7

**DOI:** 10.1259/bjro.20220016

**Published:** 2022-05-02

**Authors:** Judit Simon, Kajetan Grodecki, Sebastian Cadet, Aditya Killekar, Piotr Slomka, Samuel James Zara, Emese Zsarnóczay, Chiara Nardocci, Norbert Nagy, Katalin Kristóf, Barna Vásárhelyi, Veronika Müller, Béla Merkely, Damini Dey, Pál Maurovich-Horvat

**Affiliations:** ^1^ Medical Imaging Centre, Semmelweis University, Budapest, Hungary; ^2^ MTA-SE Cardiovascular Imaging Research Group, Heart and Vascular Center, Semmelweis University, Budapest, Hungary; ^3^ Biomedical Imaging Research Institute, Cedars-Sinai Medical Center, Los Angeles, USA; ^4^ Department of Cardiology, Medical University of Warsaw, Warsaw, Poland; ^5^ Department of Laboratory Medicine, Semmelweis University, Budapest, Hungary; ^6^ Department of Pulmonology, Semmelweis University, Budapest, Hungary

## Abstract

**Objective:**

We aimed to assess the differences in the severity and chest-CT radiomorphological signs of SARS-CoV-2 B.1.1.7 and non-B.1.1.7 variants.

**Methods:**

We collected clinical data of consecutive patients with laboratory-confirmed COVID-19 and chest-CT imaging who were admitted to the Emergency Department between September 1– November 13, 2020 (non-B.1.1.7 cases) and March 1–March 18, 2021 (B.1.1.7 cases). We also examined the differences in the severity and radiomorphological features associated with COVID-19 pneumonia. Total pneumonia burden (%), mean attenuation of ground-glass opacities and consolidation were quantified using deep-learning research software.

**Results:**

The final population comprised 500 B.1.1.7 and 500 non-B.1.1.7 cases. Patients with B.1.1.7 infection were younger (58.5 ± 15.6 vs 64.8 ± 17.3; *p* < .001) and had less comorbidities. Total pneumonia burden was higher in the B.1.1.7 patient group (16.1% [interquartile range (IQR):6.0–34.2%] *vs* 6.6% [IQR:1.2–18.3%]; *p* < .001). In the age-specific analysis, in patients <60 years B.1.1.7 pneumonia had increased consolidation burden (0.1% [IQR:0.0–0.7%] *vs* 0.1% [IQR:0.0–0.2%]; *p* < .001), and severe COVID-19 was more prevalent (11.5% vs  4.9%; *p* = .032). Mortality rate was similar in all age groups.

**Conclusion:**

Despite B.1.1.7 patients were younger and had fewer comorbidities, they experienced more severe disease than non-B.1.1.7 patients, however, the risk of death was the same between the two groups.

**Advances in knowledge:**

Our study provides data on deep-learning based quantitative lung lesion burden and clinical outcomes of patients infected by B.1.1.7 VOC. Our findings might serve as a model for later investigations, as new variants are emerging across the globe.

## Introduction

A new lineage of the SARS-CoV-2 virus named B.1.1.7 was identified in the United Kingdom in December 2020. Since then, B.1.1.7 variant of concern (VOC) has been increasing in prevalence across Europe. The B.1.1.7 variant is characterized by several genetic mutations (including N501Y substitution) in the immunodominant spike protein including the receptor-binding domain.^1^ Prior investigations have suggested that B.1.1.7 is more transmissible.^1,2^ However, results regarding the effects of B.1.1.7 on pneumonia severity are inconsistent. While Dan Frampton and colleagues reported that disease severity and clinical outcomes between patients with B.1.1.7 and non-B.1.1.7 infections were similar, three other studies have shown that lineage B.1.1.7 was linked with increased mortality.^2–4^

CT plays an essential role in the assessment of disease severity as well as in the evaluation of the temporal changes of the extent of pneumonia.^5,6^ However, since not all patients with COVID-19 have complicated disease, chest CT should be reserved for patients with specific clinical indications, as recommended by the American College of Radiology.^7^

During the early exudative phase of COVID-19 pneumonia, ground-glass opacity (GGO) in the lower lobes is the main characteristic CT abnormality progressing into consolidation at later stages of the disease course.^6,8,9^ To the best of our knowledge, no prior studies have investigated the differences in quantitative metrics and radiomorphological appearance of pneumonia between B.1.1.7 and non-B.1.1.7 SARS-CoV-2 infections.

We aimed to investigate the differences in demographic, clinical, and laboratory data between B.1.1.7 and non-B.1.1.7 SARS-CoV-2 infections. Moreover, we assessed whether there was a difference in CT-derived quantitative measures between the two lineages. In a secondary analysis, we aimed to determine the age-specific differences in B.1.1.7 and non-B.1.1.7 SARS-CoV-2 infections. In addition, we sought to investigate the clinical outcomes in the patient groups.

## Methods and materials

### Study design and setting

This analysis included consecutive patients with positive reverse transcription polymerase chain reaction (RT-PCR) result for SARS-CoV-2 who underwent non-contrast chest-CT between September 1–November 13, 2020 (500 non-B.1.1.7 cases) and March 1–March 18, 2021 (500 B.1.1.7 cases). The only exclusion criteria was missing clinical data. These date intervals were selected due to the dominance of non-B.1.1.7 and B.1.1.7 variants. A PCR specific for N501Y mutation of SARS-CoV-2 spike protein gene (Omixon Ltd, Budapest) revealed that during March 1–March 18, 2021 all the patients were infected with the B.1.1.7 variants. A retrospective analysis of 150 samples stored from the period between September 1 and November 13, 2020 indicated that N501Y mutation was absent. The clinical trial protocol was approved by the Ethics Committee of our University (SE RKEB: 256/2020).

### Clinical severity and definitions

Clinical severity was graded according to the clinical progression scale of the World Health Organization (WHO).^10^ The scale provides a measure of illness severity across a range from 0 (not infected) to 10 (dead). Based on the WHO score, the patients were classified into four categories according to their outcome: (a) mild disease included asymptomatic or symptomatic cases without the need for in-hospital treatment; (b) moderate disease was defined as hospitalized patients with or without oxygen therapy by mask or nasal prongs; (c) severe disease included hospitalized patients who needed oxygen by non-invasive ventilation or high flow, intubation, mechanical ventilation, vasopressor and/or extracorporeal membrane oxygenation and (d) death during in-hospital stay.

Potential confounders included age, sex, body mass index (BMI), hypertension, diabetes mellitus, hyperlipidemia, smoking status, history of chronic lung disease (including asthma, chronic obstructive pulmonary disease, and/or obstructive sleep apnea), heart failure, myocardial infarction, chronic kidney disease (defined as estimated glomerular filtration rate of less than 60 ml/min/1.73 m^2^) and immunodeficiency (defined as the history of cancer, organ transplantation and/or patients on disease-modifying antirheumatic drugs or glucocorticoids before hospital admission). Self-reported duration and characteristics of symptoms were also collected. Serum laboratory values were obtained at hospital admission.

### Scan protocol and image reconstruction

Non-contrast enhanced chest-CT scans were acquired using a 128-slice CT scanner (Philips Incisive, Philips Healthcare, Best, The Netherlands) in supine position during an inspiratory breath-hold. The CT acquisition protocol included a peak tube voltage of 120 kV, automatic tube current modulation (300–500 mAs), and slice thickness of 1 mm, reconstruction increment 0.85 with a collimation of 64 × 0.625. Infection control and protection were taken into account in all cases. Images were reconstructed using standard lung filters. For patients with serial chest-CT examinations, only admission scans were included in the analysis.

### CT image analysis

Standard lung window [width of 1500 Hounsfield unit (HU) and level of −400 HU] was used for image analysis. Lung abnormalities including GGO, consolidation, and pleural effusion were quantified with a deep-learning research software (LungQuant v. 1.0, Cedars-Sinai Medical Center, Los Angeles, CA).^11^ First GGO and high-opacities (comprising consolidation and pleural effusion) were segmented using convolutional Long Short-Term Memory (ConvLSTM) network. The acquired lesion masks were then edited when necessary to differentiate consolidation from pleural effusion with semi-automated brush-like tool; the boundaries of which were delimited by a region-growing algorithm. Adaptive thresholds were used, defined by a fixed window around the attenuation of the pixel clicked by the operator. Lobe segmentation was computed using the pulmonary mask and a second deep-learning model trained with the Lung Tissue Research Consortium data set. The right lung was divided into upper, middle and lower lobes by the horizontal and oblique fissures and the left lung was divided into upper and lower lobes by the oblique fissure.

GGO was defined as hazy opacities that do not obscure the underlying bronchial or vascular structures, consolidation as opacification obscuring the underlying bronchial and vascular structures and pleural effusion as a fluid collection in the pleural cavity. Chronic lung abnormalities such as emphysema or fibrosis were excluded from segmentation. Volumes of lesion components and total lesion volumes were automatically calculated by the software. Lesion burdens were calculated as total lesion volume/total lung volume x 100%. The attenuation of lesion components was defined as the mean attenuation in HUs of the total lesion volume.

### Statistical analysis

Data were tested for normality with Shapiro–Wilk test. Categorical variables were expressed as absolute numbers and percentages and continuous variables were expressed as mean ± standard deviation or median with interquartile range (IQR), as appropriate. Univariate comparisons were done with χ^2^ or Fisher’s exact tests and continuous variables were compared using Student’s *t*-test or Wilcoxon-Mann–Whitney rank-sum test, as appropriate. In order to assess the associations of clinical factors and type of SARS-CoV-2 lineage with CT-derived pneumonia severity, uni- and multivariate linear regression analyses were performed. As established by the prior studies, the following clinical predictors were entered into multivariate model: age, sex, BMI, hypertension, diabetes mellitus, hyperlipidemia, smoking status, history of chronic lung disease, heart failure, myocardial infarction, coronary artery revascularization, chronic kidney disease, immunodeficiency, serum C-reactive protein level and type of SARS-CoV-2 lineage (B.1.1.7 or non-B.1.1.7).^12^ A two-sided *p*-value <.05 was considered statistically significant. All analyses were performed using R (v. 4.0.2).

### Role of the funding source

The funding source had no influence on study design, on the collection, analysis, and interpretation of data, on the writing of the manuscript, or on the decision to submit the paper for publication.

## Results

### Demographic and baseline clinical data

**Figure 1.**
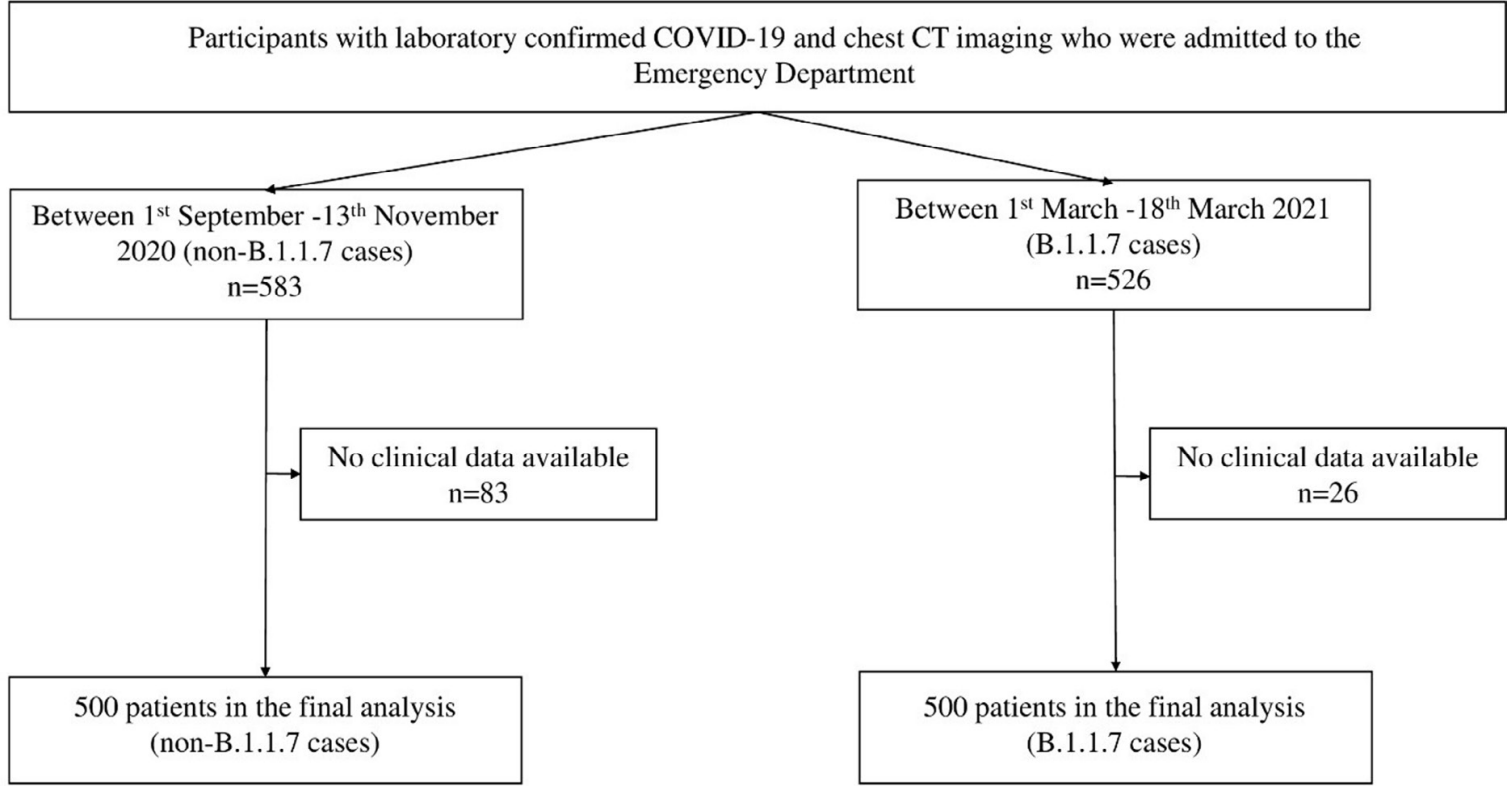
Flowchart of included patients in the study.

Altogether 1000 patients (500 with B.1.1.7 and 500 with non-B.1.1.7 infection) were included in our analysis. Flowchart of patient inclusion can be seen in Figure 1. Patients with B.1.1.7 were younger (58.5 ± 15.6 years *vs* 64.8 ± 17.3 years; *p* < .001) and had less comorbidities such as hypertension (53.9% vs  66.7%; *p* < .001), diabetes mellitus (21.1% vs  28.7%; *p* = .007), hyperlipidemia (9.3% vs  18.5%; *p* < .001), lung disease (7.8% vs  14.5%; *p* = .001), heart failure (8.5% vs  13.7%; *p* = .011), myocardial infarction (3.6% vs  7.5%; *p* = .012), chronic kidney disease (3.8% vs  11.9%; *p* < .001) and immunodeficiency (8.2% vs  18.6%; *p* < .001). There was no difference in the duration of symptoms to the time of chest-CT (7 days [IQR: 4–7 days] *vs* 6 days [IQR: 4–8 days]; *p* = .817). Patients with B.1.1.7 had a significantly greater number of symptoms such as fever (66.7% vs  43.7%; *p* < .001), dyspnea (54.0% vs  38.6%; *p* < .001), dry cough (55.4% vs  40.0%; *p* < .001) and hemoptosis (4.2% vs  0.8%; *p* = .001), while those with non-B.1.1.7 infection had greater number of cases with sputum production (5.4% vs  10.0%; *p* = .011), loss of smell (2.4% vs  8.1%; *p* < .001), loss of taste (2.8% vs  8.3%; *p* < .001) and muscle and/or joint pain (7.7% vs  12.6%; *p* = .014). Regarding the laboratory parameters patients with B.1.1.7 variant had significantly higher levels of C-reactive protein (89.4 mg l^−1^ [36.5–146.1 mg l^−1^] *vs* 60.7 mg l^−1^ [15.6–123.0 mg l^−1^]; *p* < .001). Detailed data on demographic and baseline clinical parameters are reported in Table 1.

**Table 1.** Demographic, baseline clinical and laboratory data

	Second wave (*n* = 500)	Third wave (*n* = 500)	*p*
Age (years)	64.8 ± 17.3	58.5 ± 15.6	<.001
Male sex, n (%)	268 (53.6)	287 (57.5)	.237
Body mass index (kg/m2)	29.0 ± 6.4	30.8 ± 6.1	.020
Hypertension, n (%)	330 (66.7)	268 (53.9)	<.001
Diabetes mellitus, n (%)	142 (28.7)	105 (21.1)	.007
Hyperlipidemia, n (%)	91 (18.5)	46 (9.3)	<.001
Smoking ever, n (%) (*n* = 301, 248)	101 (33.6)	74 (29.8)	.402
History of lung disease, n (%)	72 (14.5)	39 (7.8)	.001
History of heart failure, n (%)	68 (13.7)	42 (8.5)	.011
History of myocardial infarction, n (%)	37 (7.5)	18 (3.6)	.012
Chronic kidney disease, n (%)	59 (11.9)	19 (3.8)	<.001
Immunodeficiency, n (%)	92 (18.6)	41 (8.2)	<.001
Onset of symptoms to chest-CT (days)	6 (4–8)	7 (4–7)	.817
Fever, n (%)	216 (43.7)	331 (66.7)	<.001
Chills, n (%)	13 (2.6)	21 (4.2)	.231
Fatigue, n (%)	140 (28.5)	156 (31.5)	.338
Dyspnea, n (%)	190 (38.6)	268 (54.0)	<.001
Dry cough, n (%)	197 (40.0)	275 (55.4)	<.001
Sputum production, n (%)	49 (10.0)	27 (5.4)	.011
Hemoptosis, (%)	4 (0.8)	21 (4.2)	.001
Sore throat, n (%)	16 (3.3)	18 (3.6)	.880
Loss of smell, n (%)	40 (8.1)	12 (2.4)	<.001
Loss of taste, n (%)	41 (8.3)	14 (2.8)	<.001
Muscle or joint pain, n (%)	62 (12.6)	38 (7.7)	.014
Nausea, vomiting, n (%)	56 (11.4)	45 (9.1)	.274
Diarrhea, n (%)	46 (9.3)	46 (9.3)	1.000
Lymphopenia, n (%)	203 (40.9)	273 (54.8)	<.001
Lymphocytes, % (*n* = 495, 495)	17.6 (11.7–26.0)	15.6 (10.6–21.5)	.304
LDH, U/L (*n* = 416, 400)	255.5 (190.0–346.0)	378.5 (287.8–517.0)	<.001
C-reactive protein, mg/L (*n* = 492, 496)	60.7 (15.6–123.0)	89.4 (36.5–146.1)	<.001
Ferritin, ng/mL (*n* = 406, 356)	464.5 (233.0–901.0)	946.0 (474.8–1563.5)	<.001
Prothrombin time, sec (*n* = 370, 352)	9.0 (8.5–9.9)	8.8 (8.3–9.4)	<.001
D-dimer, mg/mL (*n* = 364, 335)	1.1 (0.6–2.6)	1.0 (0.6–1.6)	.126
Troponin, ng/L (*n* = 350, 376)	16.0 (8.0–37.8)	11.0 (8.0–23.0)	<.001
Creatine phosphokinase, U/L (*n* = 319, 349)	84.0 (36.5–178.5)	162.0 (85.0–384.0)	<.001
WHO Category			
Mild	49 (9.8)	58 (11.6)	.015
Moderate	307 (61.4)	304 (60.8)	
Severe	25 (5.0)	46 (9.2)	
Death	119 (23.8)	92 (18.4)	

LDH, lactate dehydrogenase; WHO, World Health Organization.

### Chest-CT findings

The prevalence of GGO and consolidation was similar between the two groups, while pleural effusion was more common in patients with non-B.1.1.7 infection (7.6% vs  24.2%; *p* < .001). Bilateral pneumonia was more common in B.1.1.7 patients (96.8% vs  91.2%; *p* < .001). Regarding the lobar distribution, pneumonia was depicted more often in each of the five lung lobes in B.1.1.7 infection with the biggest differences in the right upper (96.2% vs  88.4%; *p* < .001), right medial (92.6% vs  77.4%; *p* < .001) and left upper (95.2% vs  87.0%; *p* < .001) lobes. Accordingly, pneumonia affecting all five lung lobes was more common in patients with B.1.1.7 lineage (90.8% vs  71.2%; *p* < .001).

Patients with B.1.1.7 infection had higher total lesion burden (16.1% [IQR: 6.0–34.2%] *vs* 6.6% [IQR: 1.2–18.3%]; *p* < .001) and GGO burden (14.2 [5.5–32.6%] *vs* 3.9% [IQR: 0.6–12.6%; *p* < .001). The mean attenuation of total lung lesion (−451.8 ± 177.6 HU *vs* −482.8 ± 119.0 HU; *p* = .001), GGO (−511.9 ± 88.3 HU *vs* −532.5 ± 90.8 HU; *p* < .001) and consolidation (−124.7 ± 89.9 HU *vs* −147.3 ± 115.4 HU; *p* = .002) were higher in infections caused by the B.1.1.7 lineage. Detailed results on the chest-CT findings can be seen in Supplementary Table 1.

### Age-specific differences in the radio-morphological features and severity of pneumonia

To examine the variant-specific differences in severity as stratified by age, we divided the patients into three age groups: <60 years (*n* = 423; 61.5% with B.1.1.7), 60–75 years (*n* = 314; 48.4% with B.1.1.7) and >75 years (*n* = 263; 33.5% with B.1.1.7). Regarding the prevalence of lung abnormalities, we found differences between the two lineages only among younger patients (<60 years). In this group, GGO without consolidation was more common in patients with non-B.1.1.7 infection (26.5% vs  38.7%; *p* = .012), while GGO and consolidation was more prevalent in those with B.1.1.7 (70.4%vs 50.9%; *p* < .001). Pleural effusion was more common in non-B.1.1.7 infection in all three age groups. Bilateral pneumonia was more common in B.1.1.7 infection across the age groups, but the difference proved to be statistically significant only in patients <60 years (94.6% vs  79.1%; *p* < .001). As for lobar involvement, pneumonia affecting all five lung lobes was more prevalent in patients under 75 years with B.1.1.7 (86.9% vs 57.7% in patients <60 years and 96.7 vs 77.2% in those between 60 and 75 years; both *p* < .001), while in the oldest patient group the difference was not significant.

**Figure 2.**
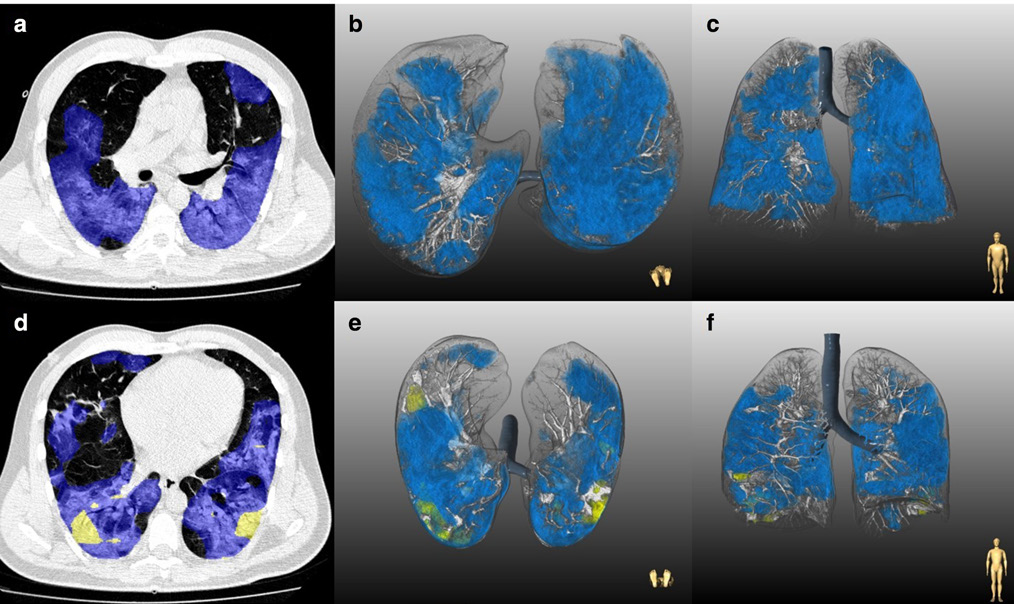
Representative examples of chest-CT findings in patients with non-B.1.1.7 (**A–C**) and B.1.1.7 (**D–F**) infection. A–C: Chest-CT study of a 28-year-old male with non-B.1.1.7 pneumonia. The patient had obesity, hypertension and diabetes mellitus. At the time of hospital admission, oxygen saturation was 88% and patient had fever, fatigue, dyspnea, loss of smell and taste, nausea and vomiting. Patient was discharged after an uncomplicated 10-day hospital admission. A: Axial slice depicts bilateral GGOs (blue). Lesion quantification revealed a GGO burden of 42.8% without consolidation. B: Three-dimensional lung renderings in axial and C: coronal planes. D–F: Chest-CT study of a 45-year-old male with B.1.1.7 pneumonia. The patient had normal body mass index and no comorbidities. At the time of hospital admission oxygen saturation was 86% and patient had fever, cough and dyspnea. Patient needed admission to intensive care unit with mechanical ventilation, vasopressor therapy, extracorporeal membrane oxygenation and died 14 days after hospital admission. A: Axial slice demonstrating 40.4% total lesion burden with 39.2% GGO burden (blue) and 1.2% consolidation burden (yellow) at the time of hospital admission. B: Three-dimensional lung renderings in axial and C: coronal planes. GGO, ground-glass opacity.

**Figure 3.**
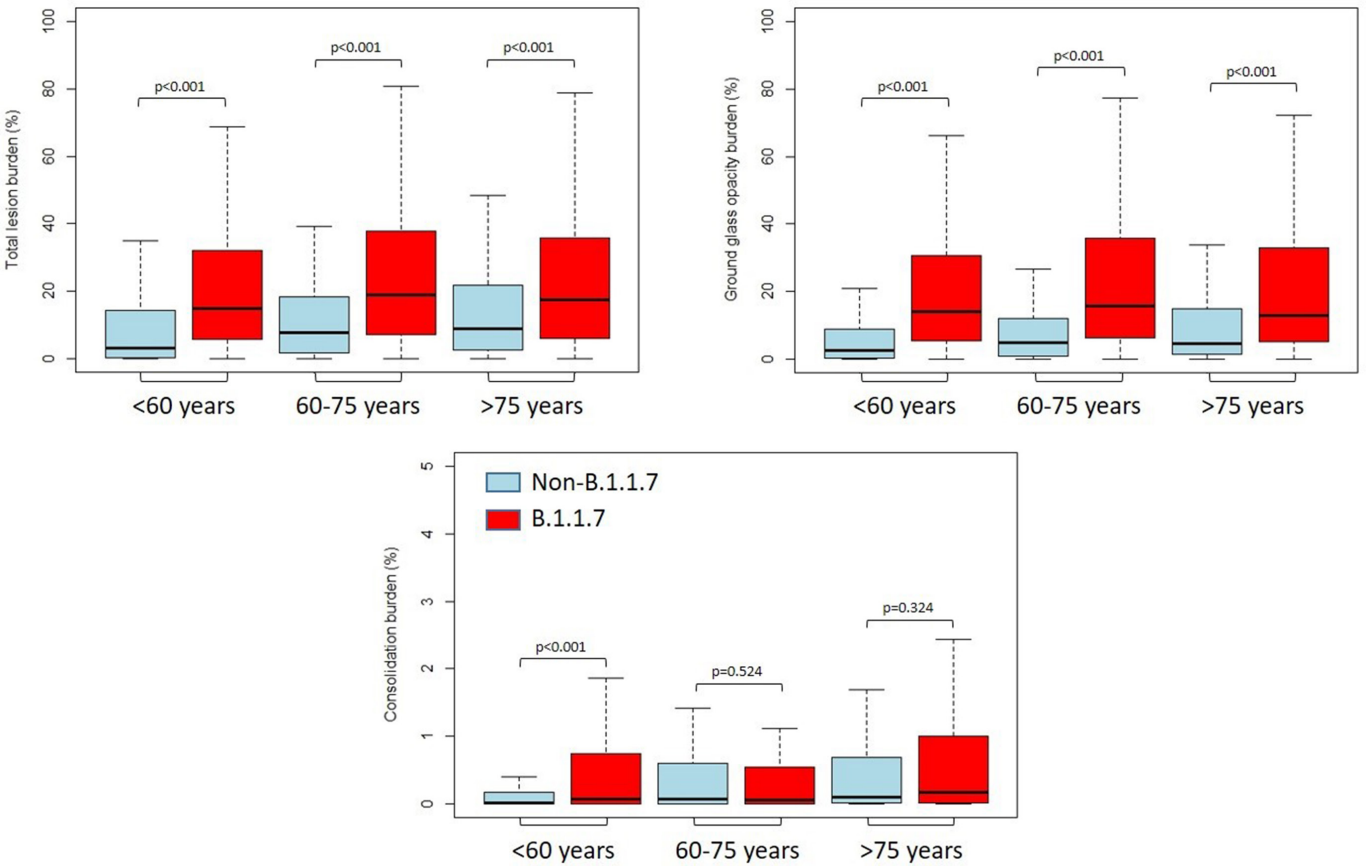
Burden of lung abnormalities between non-B.1.1.7 and B.1.1.7 infections Box plots show median, 25th–75th interquartile range with minimum and maximum values

Quantitative lung features also differed significantly across the age groups. While total lesion burden (14.7% [IQR: 5.5–32.0%] *vs* 3.1% [IQR: 0.2–14.2%] in patients <60 years, 18.9% [IQR: 7.2–37.0%] *vs* 7.7% [IQR: 1.8–18.0%] in patients 60–75 years and 17.4% [6.0–35.6%] *vs* 8.9% [IQR: 2.4–21.7%]; all *p* < .001) and GGO burden (13.9% [IQR: 5.4–30.4%] *vs* 2.6% [IQR: 0.1–8.9%] in patients <60 years, 15.7% [IQR: 6.3–35.7%] *vs* 4.8% [IQR: 0.8–11.9%] in patients 60–75 years and 12.7% [IQR: 5.2–32.9%] *vs* 4.5% [IQR: 1.2–14.8%] in those >75 years; all *p* < .001) were significantly higher in B.1.1.7 pneumonia in all three age groups, consolidation burden significantly differed only in those patients <60 years (0.1% [IQR: 0.0–0.7] *vs* 0.1% [IQR:0.0–0.2%]; *p* < .001). The mean attenuation of total lung lesion (−482.4 ± 102.6 HU *vs* −492.5 ± 160.9 HU; *p* = .041) and GGO (−502.0 ± 83.8 HU *vs* −541.4 ± 103.8 HU; *p* < .001) were significantly higher in B.1.1.7 pneumonia, but only in those <60 years. Representative cases of CT findings are shown in Figure 2. Detailed data on CT parameters of non-B.1.1.7 and B.1.1.7 pneumonia across the age groups are reported in Table 2 and Figure 3.

**Table 2.** Characteristics and quantitative measures of lung lesions, as stratified by age

	<60 years (*n* = 423)			60–75 years (*n* = 314)			<75 years (*n* = 263)		
	Non-B.1.1.7 (*n* = 163)	B.1.1.7 (*n* = 260)	p	Non-B.1.1.7 (*n* = 162)	B.1.1.7 (*n* = 152)	p	Non-B.1.1.7 (*n* = 175)	B.1.1.7 (*n* = 88)	p
Lung abnormality									
Only ground-glass opacities, n (%)	63 (38.7)	69 (26.5)	.012	54 (33.3)	52 (34.2)	.964	35 (20.0)	17 (19.3)	1.000
Only consolidation, n (%)	1 (0.6)	0 (0.0)	.814	1 (0.6)	0 (0.0)	1.000	35 (20.0)	17 (19.3)	1.000
Ground-glass opacities and consolidation, n (%)	83 (50.9)	183 (70.4)	<.001	106 (65.4)	99 (65.1)	1.000	139 (79.4)	70 (79.5)	1.000
Pleural effusion, n (%)	17 (10.4)	10 (3.8)	.013	44 (27.2)	13 (8.5)	<.001	60 (34.3)	15 (17.0)	.005
None, n (%)	14 (8.6)	7 (2.7)	.007	1 (0.6)	1 (0.6)	1.000	1 (0.6)	1 (1.1)	1.000
Laterality									
Unilateral, n (%)	20 (12.3)	7 (2.7)	<.001	6 (3.7)	0 (0.0)	.047	2 (1.1)	0 (0.0)	.799
Only right, n (%)	13 (8.0)	5 (1.9)	.006	4 (2.5)	0 (0.0)	.148	1 (0.6)	0 (0.0)	1.000
Only left, n (%)	7 (4.3)	2 (0.8)	.036	2 (1.2)	0 (0.0)	.506	1 (0.6)	0 (0.0)	1.000
Bilateral, n (%)	129 (79.1)	246 (94.6)	<.001	155 (95.7)	151 (99.3)	.089	172 (98.3)	87 (98.9)	1.000
Lobar distribution									
Right upper lobe, n (%)	125 (76.7)	244 (93.8)	<.001	148 (91.4)	151 (99.3)	.002	169 (96.6)	86 (97.7)	.893
Right medial lobe, n (%)	104 (63.8)	229 (88.1)	<.001	136 (84.0)	148 (97.4)	<.001	147 (84.0)	86 (97.7)	.002
Right lower lobe, n (%)	137 (84.0)	250 (96.2)	<.001	155 (95.7)	151 (99.3)	.089	170 (97.1)	87 (98.9)	.657
Left upper lobe, n (%)	120 (73.6)	244 (93.8)	<.001	149 (92.0)	149 (98.0)	.029	166 (94.9)	83 (94.3)	1.000
Left lower lobe, n (%)	134 (82.2)	247 (95.0)	<.001	155 (95.7)	151 (99.3)	.089	167 (95.4)	85 (96.6)	.906
Lobar involvement									
One lobe, n (%)	14 (8.6)	2 (0.7)	<.001	3 (1.9)	0 (0.0)	<.001	2 (1.1)	0 (0.0)	.085
Two lobes, n (%)	16 (9.8)	5 (1.9)		4 (2.5)	0 (0.0)		1 (0.6)	0 (0.0)	
Three lobes, n (%)	8 (4.9)	8 (3.1)		13 (8.0)	1 (0.7)		6 (3.4)	2 (2.3)	
Four lobes, n (%)	20 (12.3)	12 (4.6)		17 (10.5)	3 (2.0)		28 (16.0)	4 (4.5)	
Five lobes, n (%)	94 (57.7)	226 (86.9)		125 (77.2)	147 (96.7)		137 (78.3)	81 (92.0)	
Lung lesion volume (ml)									
Total	107.9 (4.4–493.4)	526.8 (219.3–1110.5)	<.001	307.8 (61.5–673.6)	715.3 (294.1–1459.6)	<.001	303.7 (75.6–724.9)	618.0 (226.7–1229.6)	<.001
Ground-glass opacities	92.0 (3.0–345.1)	490.5 (202.5–1049.7)	<.001	216.4 (34.6–488.4)	654.2 (248.6–1378.6)	<.001	163.7 (32.0–487.2)	507.0 (169.0–1141.0)	<.001
Consolidation	0.2 (0.0–6.3)	2.5 (0.1–26.2)	<.001	3.4 (0.0–23.7)	1.9 (0.1–18.3)	.740	3.6 (0.3–21.0)	5.0 (0.4–36.1)	.337
Effusion	0.0 (0.0–0.0)	0.0 (0.0–0.0)	.006	0.0 (0.0–21.4)	0.0 (0.0–0.0)	<.001	0.0 (0.0–49.3)	0.0 (0.0–0.0)	.008
Lungs lesion burden (%)									
Total	3.1 (0.2–14.2)	14.7 (5.5–32.0)	<.001	7.7 (1.8–18.0)	18.9 (7.2–37.0)	<.001	8.9 (2.4–21.7)	17.4 (6.0–35.6)	<.001
Ground-glass opacities	2.6 (0.1–8.9)	13.9 (5.4–30.4)	<.001	4.8 (0.8–11.9)	15.7 (6.3–35.7)	<.001	4.5 (1.2–14.8)	12.7 (5.2–32.9)	<.001
Consolidation	0.1 (0.0–0.2)	0.1 (0.0–0.7)	<.001	0.1 (0.0–0.6)	0.1 (0.0–0.5)	.524	0.1 (0.0–0.7)	0.2 (0.0–0.9)	.324
Effusion	0.0 (0.0–0.0)	0.0 (0.0–0.0)	.006	0.0 (0.0–0.6)	0.0 (0.0–0.0)	<.001	0.0 (0.0–1.6)	0.0 (0.0–0.0)	.008
Lungs lesion mean attenuation (HU)									
Total	−492.5 ± 160.9	−482.4 ± 102.6	.041	−449.7 ± 184.9	−500.0 ± 128.3	.062	−417.1 ± 178.6	−454.1 ± 140.5	.262
Ground-glass opacities	−541.4 ± 103.8	−502.0 ± 83.8	<.001	−536.1 ± 83.2	−531.3 ± 91.3	.969	−521.2 ± 84.2	−507.2 ± 91.3	.283
Consolidation	−152.7 ± 132.3	−115.6 ± 85.3	.117	−149.0 ± 104.6	−144.8 ± 99.2	.686	−142.0 ± 111.8	−116.6 ± 81.9	.065
Effusion	−22.6 ± 34.6	−15.9 ± 21.7	.712	−30.9 ± 36.7	−23.1 ± 29.3	.645	−38.8 ± 41.0	−31.3 ± 38.2	.592

We also examined if the type of lineage was significantly associated with the pneumonia burden. In the univariate analysis, B.1.1.7 variant of concern was linked with higher total lung lesion burden in all three age groups (ß: 12.6% (95%CI: 8.9–16.3%) in patients <60 years, ß: 9.5% (95%CI: 5.2–13.8%) in patients 60–75 years and ß: 8.1% (95%CI: 3.2–12.9%) in those >75 years; all *p* < .05). After adjustment for baseline demographic and clinical data, B.1.1.7 lineage was significantly associated with increased pneumonia burden only in patients <60 years [ß: 15.0% (95%CI: 6.7–23.4%); *p* < .001]. Results of the uni- and multivariate linear regression analyses can be seen in Table 3.

**Table 3.** Association between type of SARS-CoV-2 variant and total lung lesion burden, as stratified by age

Outcome: total lung lesion burden (%)		<60 years (*n* = 423)		60–75 years (*n* = 314)		<75 years (*n* = 263)	
		ß (95% CI)	p	ß (95% CI)	p	ß (95% CI)	p
Unadjusted	Non-B.1.1.7 lineage	Ref	…	Ref	…	Ref	…
	B.1.1.7 lineage	12.6 (8.9–16.3)	<.001	9.5 (5.2–13.8)	<.001	8.1 (3.2–12.9)	.001
Adjusted	Non-B.1.1.7 lineage	Ref	…	Ref	…	Ref	…
	B.1.1.7 lineage	15.0 (6.7–23.4)	<.001	8.1 (-0.25–16.5)	.057	−0.47 (-12.5–11.6)	.938

BMI, body mass index.

a Adjusted for sex, BMI, hypertension, diabetes mellitus, hyperlipidemia, smoking status, history of chronic lung disease, heart failure, myocardial infarction, chronic kidney disease, immunodeficiency and serum C-reactive protein level.

### Clinical severity

**Figure 4.**
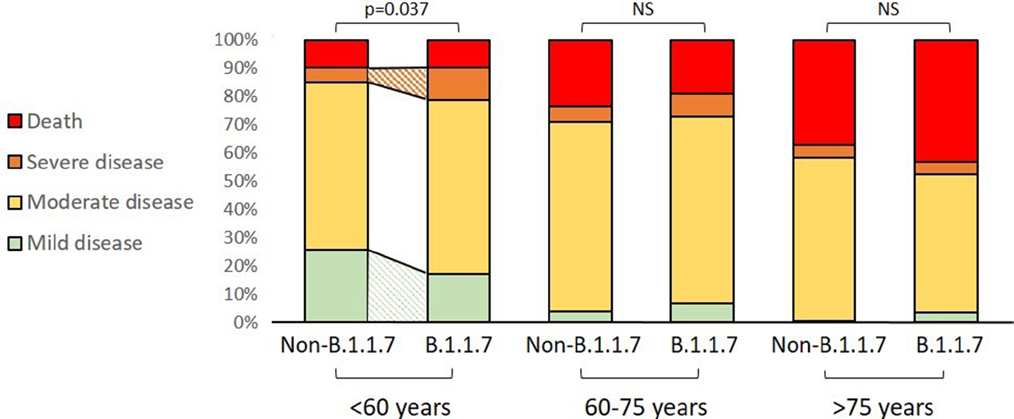
COVID-19 severity across age groups and by SARS-CoV2 lineage type.

Mortality rate was similar in all age groups (9.6% vs  9.8% in patients <60 years; 19.1 vs 23.5% in those 60–75 years and 43.2 vs 37.1% in patients >70 years; all *p* > .05). When comparing the clinical severity of COVID-19 between the two lineages, we have observed differences only in patients <60 years, where B.1.1.7 variant of concern was linked to a lower proportion of mild (17.3% vs  25.8%; *p* = .048) and a higher proportion of severe disease (11.5% vs  4.9%; *p* = .032). In the other two age groups, there were no differences in the severity of illness between the lineages. Data on the age-specific outcome stratified by type of SARS-CoV-2 variant can be seen in Table 4 and Figure 4.

**Table 4.** Age-specific severity of COVID-19 as stratified by type of SARS-CoV-2 lineage

	<60 years (*n* = 423)			60–75 years (*n* = 314)			>75 years (*n* = 263)		
	Non-B.1.1.7 (*n* = 163)	B.1.1.7 (*n* = 260)	p	Non-B.1.1.7 (*n* = 162)	B.1.1.7 (*n* = 152)	p	Non-B.1.1.7 (*n* = 175)	B.1.1.7 (*n* = 88)	p
Mild disease, n (%)	42 (25.8)	45 (17.3)	.037	6 (3.7)	10 (6.6)	.453	1 (0.6)	3 (3.4)	.214
Moderate disease, n (%)	97 (59.5)	160 (61.5)		109 (67.3)	101 (66.4)		101 (57.7)	43 (48.9)	
Severe disease, n (%)	8 (4.9)	30 (11.5)		9 (5.5)	12 (7.9)		8 (4.6)	4 (4.5)	
Death, n (%)	16 (9.8)	25 (9.6)		38 (23.5)	29 (19.1)		65 (37.1)	38 (43.2)	

## Discussion

In our study population of 1000 COVID-19 patients (50% with B.1.1.7 infection), B.1.1.7 infection was not associated with increased risk of death as compared to the non-B.1.1.7 infection. However, B.1.1.7. was associated with increased total pneumonia burden and GGO burden, and higher lung lesion attenuation as compared to non-B.1.1.7 lineage. Moreover, in patients <60 years, those with B.1.1.7 pneumonia had increased consolidation burden, and severe COVID-19 was more prevalent. The B.1.1.7 lineage was an independent predictor of pneumonia burden only in patients < 60 years of age.

Previous studies have reported that B.1.1.7 is more transmissible, as compared to other variants.^1,2^ However, results on the effect of this variant of concern on the severity of COVID-19 are less certain and the majority of these datasets do not include detailed data on the potential confounders and mediators of clinical outcome. In our study, granular patient data have been collected including demographic, baseline clinical, and laboratory values. Previous studies have shown that B.1.1.7 variant was associated with increased mortality.^3,4^ However, these studies were based on a community-based database and failed to report disease severity, while another study of 341 patients (58% with B.1.1.7 infection) reported similar disease severity and clinical outcomes between non-B.1.1.7 and B.1.1.7 infections after adjustment for age, sex, ethnicity, and comorbidities.^2^ Our results are in line with these latter conclusions in the overall population, however in younger patients (<60 years) B.1.1.7 lineage was associated with more severe COVID-19 with a similar mortality rate as the non-B.1.1.7 infection. This might be partly due to the advanced patient management over time and partly due to patient characteristics, as prevalence and number of comorbidities are strongly associated with age. Interestingly, while vaccination was not available during the first study period (non-B.1.1.7 patients), in those with B.1.1.7 infection 14 patients had previously had at least one shot of vaccination (4 in the <60 years, 4 in the 60–75 years, and 6 in the >75 years age group). These observations are in line with the European Centre for Disease Prevention and Control statistics, as less than 10 per 100 people were fully vaccinated in the age group above 60 years and less than 5 per 100 people have received both vaccine shots in the <60 years age group in Hungary as of March 1, 2021.^13^

Based on previous reports, CT is an important diagnostic tool in patients with suspected COVID-19 and combining its results with clinical and laboratory parameters could facilitate timely patient management.^14–18^ COVID-19 pneumonia manifests, even in asymptomatic patients, with rapid evolution from unilateral to bilateral GGO representing the early exudative phase within the first 5 days from the onset of symptoms followed by consolidative changes in the intraalveolar space with fibroblast proliferation and collapse of the alveoli within 1–3 weeks.^17,19^ Accordingly, the presence of consolidation has been shown to be independently associated with more severe disease and adverse outcomes.^12,20^ In our study population, however, B.1.1.7 higher total pneumonia burden was driven by increased GGO burden, as compared to non-B.1.1.7 infection.

Given recent development in the application of artificial intelligence in medical imaging, deep-learning-based approaches offer a great promise for the precise detection and prognostication of COVID-19, and several different methods have been reported recently.^21–24^ However, as of the date of this writing, no robust study on the characteristics of B.1.1.7 pneumonia evaluated from chest-CT has been reported. Here, we demonstrate distinct quantitative tomographic measures present in patients infected with B.1.1.7 VOC as assessed with the deep-learning algorithm. To our knowledge, this is the first study to investigate the radio-morphological differences between B.1.1.7 and non-B.1.1.7 infections, as quantified by a deep-learning-based algorithm in a large study population of 1000 patients (50% with B.1.1.7 infection). Moreover, detailed clinical data including demographic and laboratory parameters and comorbidities were collected in all patients. Clinical severity was graded according to the clinical progression scale of the WHO. Age-specific subanalyses were done to further investigate the differences between the two lineages.There are some limitations to be acknowledged. First, during the third wave (B.1.1.7 infections), the less severe cases might have been treated at home, and hospital admissions may have represented a more severe patient population. This might have introduced a selection bias. However, it is important to note that the admission criteria of patients with suspected COVID-19 did not change in the third wave as compared to the second wave in our country. Second, we did not analyze the progress of pneumonia during an in-hospital stay. However, the main purpose of this analysis was to study the differences between the two lineage infections. Moreover, the effect of therapy on the outcomes was not studied. Nonetheless, standard supportive care was applied with only a minority receiving targeted intervention.

COVID-19 is still a major global health problem and new SARS-CoV-2 variants raise public health concerns. Our study provides data on deep-learning-based quantitative lung lesion burden and clinical outcomes of patients infected by B.1.1.7 VOC. Our results, in combination with further studies on the effect of vaccination on the new variants are essential for the public health interventions in the COVID-19 pandemic. Our findings might serve as a model for later investigations, as new variants are emerging across the globe.
